# 4T1 Cell Membrane-Coated Pdots with NIR-II Absorption and Fluorescence Properties for Targeted Phototheranostics of Breast Tumors

**DOI:** 10.1021/acsami.4c12845

**Published:** 2024-11-21

**Authors:** Jintong Guo, Ye Liu, Xiao Liang, Zhiyi Chen, Bin Liu, Zhen Yuan

**Affiliations:** † Faculty of Health Sciences, University of Macau, Macau SAR 99999, China; ‡ Centre for Cognitive and Brain Sciences, University of Macau, Macau SAR 99999, China; § Department of Biomedical Engineering, Southern University of Science and Technology, Shenzhen, Guangdong 518055, China; ∥ Key Laboratory of Medical Imaging Precision Theranostics and Radiation Protection, College of Hunan Province, The Affiliated Changsha Central Hospital, Hengyang Medical School, University of South China, Changsha, Hunan 410004, China; ⊥ Zhujiang Hospital of Southern Medical University, Guangzhou, Guangdong 510280, China; △ Institute of Medical Imaging, Hengyang Medical School, University of South China, Hengyang, Hunan 421001, China

**Keywords:** biomimetic Pdots, photothermal therapy, fluorescence imaging, photoacoustic imaging, second near-infrared window

## Abstract

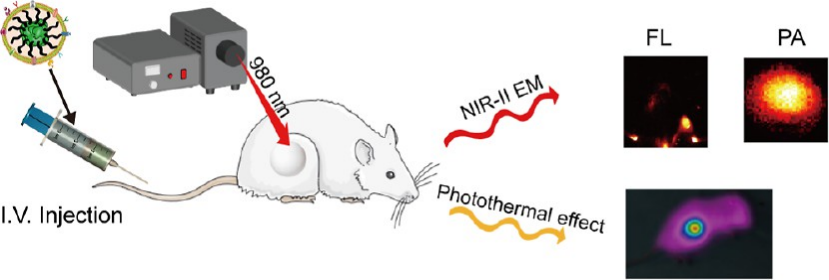

Designing highly biocompatible organic semiconducting conjugated polymer dots (Pdots) with bright fluorescence and superior absorption properties in the second near-infrared window (NIR-II: 1000–1700 nm) remains a huge challenge for tumor phototheranostics. In this study, we constructed 4T1 cell membrane-coated *m*-PBTQ4F Pdots (CPdots) with enhanced NIR-II photoacoustic (PA) and fluorescence (FL) imaging capability for NIR-II photothermal therapy (PTT) of breast tumors. Our findings demonstrated that CPdots could specifically target breast tumors, leading to enhanced tumor accumulation after systemic administration in living mice. In addition, CPdots can not only serve as contrast agents for NIR-II PA and FL imaging for improved breast tumor detection but also generate more cytotoxic heat to improve PTT efficacy. Therefore, this pilot study opens an option avenue for developing new NIR-II Pdots with homologous targeting capability for enhanced phototheranostics of breast tumors.

## 1 Introduction

To date, most of the available tumor phototheranostics strategies use nanoprobes with absorption and fluorescence emission wavelengths in the visible wavelength (400–700 nm) or first NIR window (NIR-I, 700–900 nm) for optical molecular imaging and phototherapy.^1^ However, compared to visible or NIR-I light, the second NIR window (NIR-II, 900–1880 nm)^2^ light exhibits intrinsic advantages of lower optical scattering and higher maximum permissible exposure, causing increased penetration depth and improved sensitivity for disease detection and treatment.^3^ Interestingly, various NIR-II organic or inorganic nanoprobes have been constructed to serve as contrast agents for fluorescence (FL) and photoacoustic (PA) imaging or as theragnostic agents for phototherapy.^4^ More specifically, due to the tunable optical properties, exceptional photostability, and excellent biocompatibility, Pdots represent the most attractive one among all NIR-II theragnostic agents.^5^ However, the downside of NIR-II Pdots is their low FL quantum yields (QY) due to their small energy gap in the NIR-II window.^6^ Meanwhile, Pdots with high QY might exhibit low absorption properties, demonstrating the difficulty in the development of dual-modal NIR-II PA/FL nanoprobes.^7,8^ Recently, several new strategies have been proposed to improve the concurrent fluorescence and absorption performance of Pdots. For example, Pdots with double-acceptor conjugated polymers^9^ and side chain structures^10^ were developed to carry out NIR-II FL imaging and NIR-II photothermal therapy (PTT).

Despite these advances, the in vivo biomedical application of Pdots still faces huge challenges such as early immune system recognition, rapid reticuloendothelial system clearance, and poor accumulation at tumor regions.^11^ Therefore, it is essential to develop surface-modifying Pdots for targeted and enhanced disease theragnostic.^12^ Interestingly, cell membrane-coated nanoparticles have been extensively inspected for various biomedical applications,^13^ offering biomimetic nanoplatforms with native biological capabilities.^14^ For example, biomimetic nanoparticles linked with active red blood cell membranes exhibited prolonged circulation time and reduced accelerated blood clearance.^15^ Besides, biomimetic nanoparticles have also shown the ability to pass through the blood–brain barrier and target glioma tumors for enhanced treatment.^16^ More importantly, biomimetic Pdots can be produced by using the chemical processes of electrostatic adsorption.^17^

In this study, *m*-PBTQ4F was used to produce new fluorination Pdots (*m*-PBTQ4F Pdots) that can generate highly bright FL and PA signals in the NIR-II window.^8^ For the constructed *m*-PBTQ4F Pdots, fluorination was able to elevate the fluorescence intensity of Pdots in aqueous solution, which is attributed to the nanoscale fluorous effect. According to the density functional theory, fluorination can further minimize the structural distortion between the excited and ground states, thus reducing nonradiative relaxation and significantly increasing optical absorption.^18^ Additionally, to improve the efficacy of breast cancer phototheranostics, 4T1 cancer cell membrane-coated CPdots were constructed. Further, to examine their homologous targeting ability for detecting and treating breast tumors, we first synthesized and characterized CPdots. Then in vitro and in vivo, experimental tests were conducted, illustrating that CPdots have superior capability in targeting breast tumors and can enhance tumor accumulation after systemic administration in living mice. Besides, we also discovered that CPdots can serve as a theranostic agent for concurrent NIR-II PA and FL imaging-guided NIR-II PTT of breast cancer. Therefore, CPdots, as a highly effective biomimetic nanoplatform, exhibited enhanced NIR-II phototheranostics of breast tumors with homologous targeting capability, paving a new avenue for their potential clinical applications.

## 2 Results and Discussion

### 2.1 Design and Synthesis of CPdots

**Figure 1:**
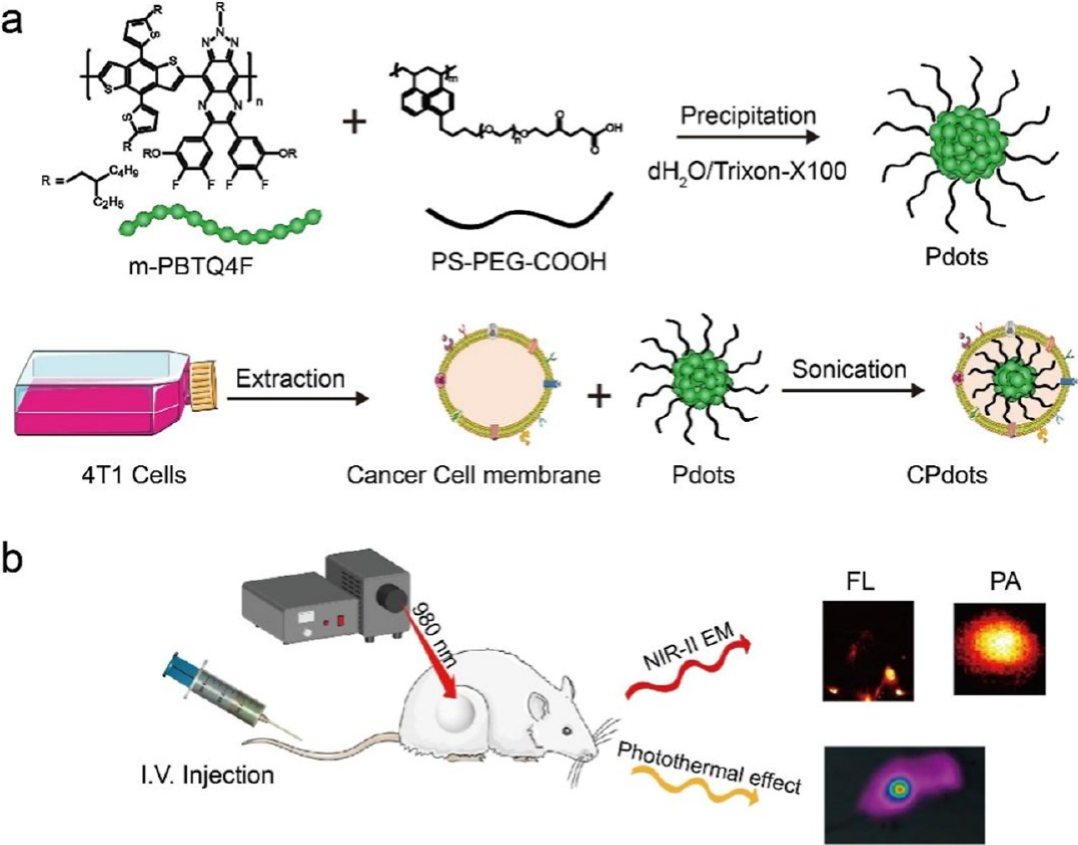
(a) Chemical structural formula of the semiconducting polymer *m*-PBTQ4F and functional polymer PS–PEG-COOH. Schematic of the preparation of *m*-PBTQ4F Pdots and CPdots. (b) Schematic of CPdots for in vivo dual-model FL/PA imaging PTT in the NIR-II window.

Pdots were prepared by blending the function polymer poly(styrene)-*graft*-poly(ethylene oxide) (PS–PEG-COOH) and a NIR-II-emitting polymer (*m*-PBTQ4F) into densely packed nanoparticles according to a previously described reprecipitation method.^8^ The chemical structures of the polymers are displayed in Figure 1a. After extracting the 4T1 cell membranes from the cancer cells, they were coated onto the surface of Pdots with 10 min of sonication to generate the CPdots (Figure 1b).

### 2.2 Characterization of CPdots

**Figure 2:**
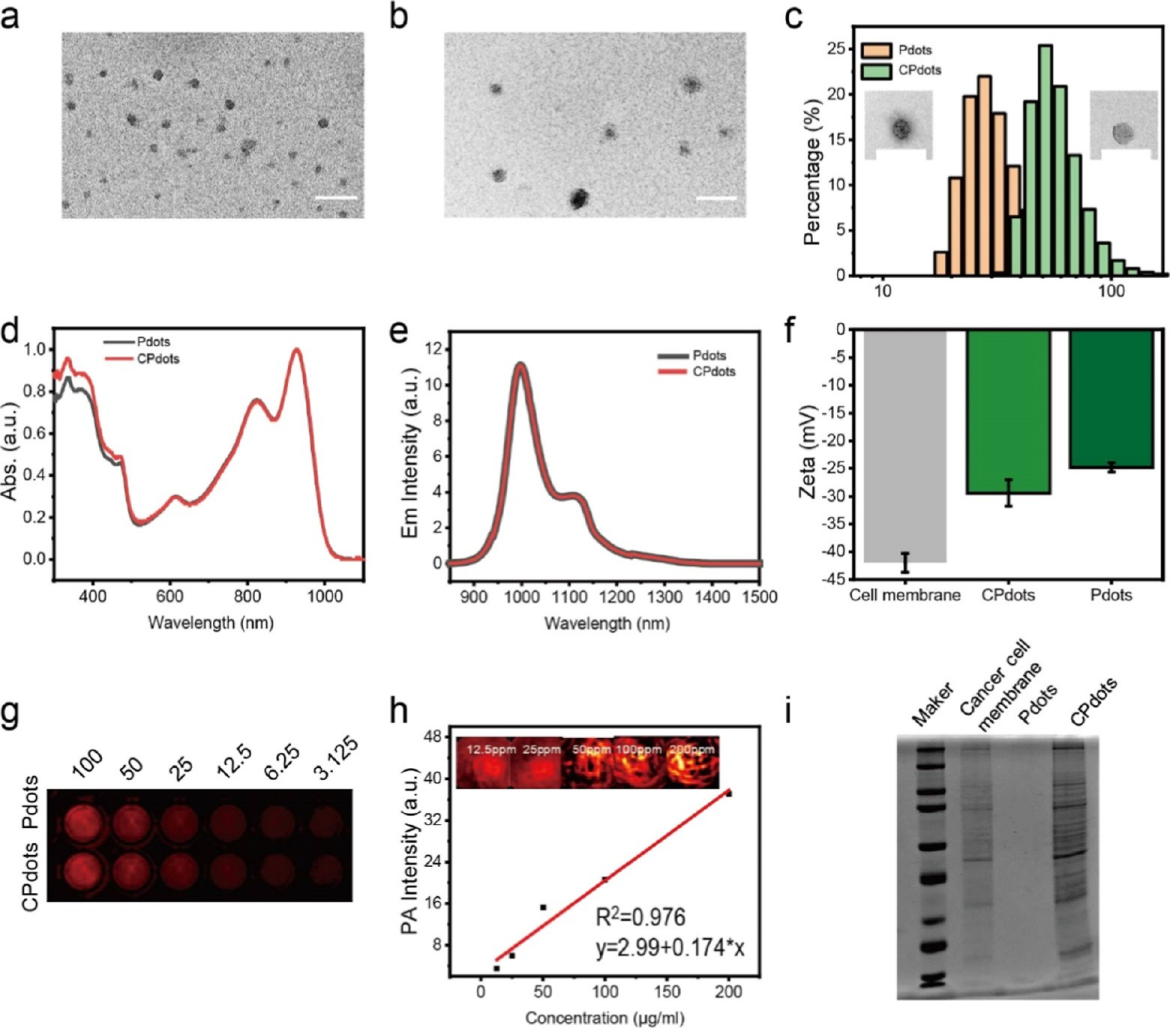
(a) TEM imaging of *m*-PBTQ4F Pdots. (b) TEM images of CPdots (scale bar: 100 nm). (c) Widths of *m*-PBTQ4F Pdots and CPdots (scale bar: 50 nm). (d) UV–vis absorption spectra of *m*-PBTQ4F Pdots and CPdots. (e) Fluorescence spectra of *m*-PBTQ4F Pdots and CPdots. (f) Zeta potential of *m*-PBTQ4F Pdots, CPdots, and 4T1 cancer cell membrane (CM), respectively. (g) Fluorescence signals of Pdots and CPdots with increased concentrations (left to right: 100, 50, 25, 12.5, 6.25, 3.125 μg/mL). (h) PA signals at 980 nm with increased Pdots concentrations. Insets are PA images of Pdots with different concentrations (μg/mL). (i) SDS-PAGE protein analysis of CM, CPdots, *m*-PBTQ4F Pdots, and protein markers. Here, CPdots denote the *m*-PBTQ4F CPdots.

Dynamic light scattering measurements and transmission electron microscopy (TEM) imaging were performed to confirm the successful coating of the cancer cell membrane onto the surface of the *m*-PBTQ4F Pdots. The analysis results are plotted in Figure 2a–c. It is discovered from Figure 2c that CPdots size increased from ∼28 to ∼45 nm as compared to that of *m*-PBTQ4F Pdots. As shown in Figure S1, the sizes of the two materials do not exhibit significant fluctuations over time. The absorption and emission spectra of CPdots and *m*-PBTQ4F Pdots were respectively plotted in Figure 2c,d. It was discovered that the *m*-PBTQ4F Pdots and CPdots showed the same bright fluorescence in the NIR-II regions under 808 nm light excitations. Meanwhile, the measured zeta potential of the cell membrane, *m*-PBTQ4F Pdots, and CPdots were −42, −25, and −30 mV, respectively (Figure 2f). All of the results demonstrate the successful modification of biofilms on the surface of *m*-PBTQ4F Pdots without compromising their optical properties. In addition, in vitro, phantom tests demonstrated that FL signals were enhanced with increased concentrations of *m*-PBTQ4F Pdots and CPdots from 3.125 to 100 μg/mL (Figure 2g); also the PA signals of Pdots from 12.5 to 200 μg/mL are shown in Figure 2h Statistical data also indicate that the fluorescence intensities of Pdots and CPdots are essentially consistent (Figure S2, Supporting Information). Subsequently, SDS-PAGE protein analysis was performed to inspect the cancer cell membrane, *m*-PBTQ4F Pdots, and CPdots, demonstrating that almost all cell membrane proteins were extensively retained on the surface of nanoparticles (Figure 2i).

### 2.3 In Vitro PTT of *m*-PBTQ4F Pdots

**Figure 3:**
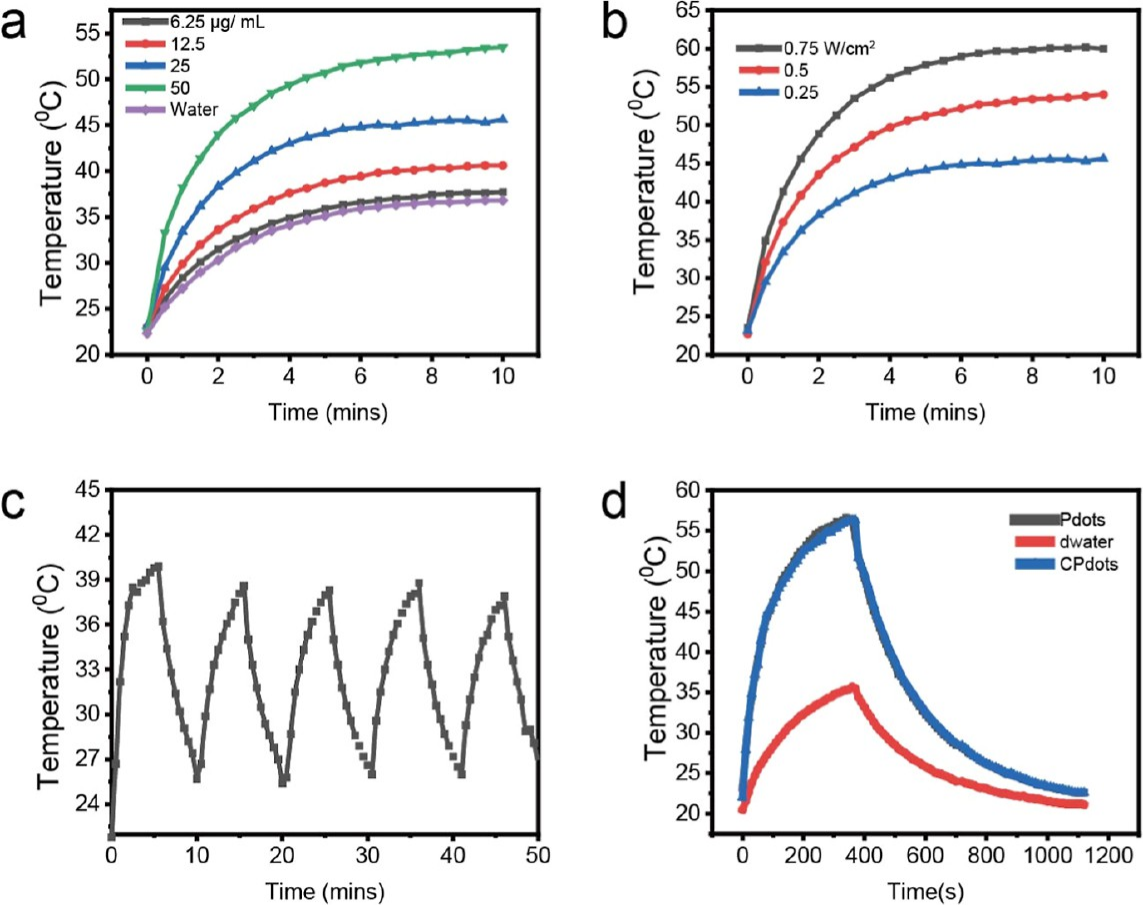
(a) Photothermal heating curves of *m*-PBTQ4F Pdot solutions with different concentrations (6.25, 12.5, 25, and 50 μg/mL) and pure water under 980 nm laser irradiation with power density of 0.5 W/cm^2^ for 10 min. (b) Photothermal heating curves of Pdots dispersions (25 μg/mL) irradiated by a 980 nm laser at various power densities (0.25, 0.5, and 0.75 W/cm^2^). (c) Temperature elevation of 25 μg/mL *m*-PBTQ4F Pdots dispersions under five on/off cycles under 980 nm laser irradiation at 0.25 W/cm^2^. (d) Photothermal performance of *m*-PBTQ4F Pdots, CPdots, and water under 980 nm laser irradiation for heating and cooling periods.

Due to their remarkable and broad absorption property in the NIR-II window, Pdots can serve as contrast agents for NIR-II PA imaging (PAI) and concurrent photothermal agents for NIR-II PTT. To assess their photothermal property, the temperature variation of *m*-PBTQ4F Pdots solution under 980 nm laser irradiation was inspected in vitro with different concentrations (6.25, 12.5, 25, and 50 μg/mL) (Figure 3a). It was discovered that a rapid temperature increase was detected in the solution, even at a relatively low concentration of 12.5 mg/mL under 5 min laser irritation. In particular, the temperature elevation of *m*-PBTQ4F Pdots (50 μg/mL) can be up to 55 °C. In addition, we also examined the photothermal properties at various laser power densities (0.25, 0.5, and 0.75 W/cm^2^). As shown in Figure 3b, *m*-PBTQ4F Pdots exhibited a strong laser-power-dependent photothermal effect under continuous irradiation.

To further evaluate the photothermal stability of *m*-PBTQ4F Pdots, their recyclable temperature evolution was inspected, and the results are displayed in Figure 3c. The temperature evolution of *m*-PBTQ4F Pdot and CPdots dispersions upon 980 nm laser irradiation was monitored over time with reversible heating–cooling cycles (Figure 3d). During the five laser on/off cycles of NIR laser irradiation, no obvious change in the highest temperature was detected, demonstrating the photostability of the *m*-PBTQ4F Pdots. To access the photothermal conversion efficiency, the *m*-PBTQ4F Pdots solution was irradiated with a 980 nm laser for 6 min until it reached a steady temperature state. Then, we stopped the laser irradiation to allow the Pdots solution to cool to an ambient temperature, with pure water serving as a negative control. The photothermal conversion efficiency of Pdots was around 52%, which was quantified based on the previous method (Figure S3 and eqs S1–S9, Supporting Information).^19^ Moreover, this PCE demonstrates significant superiority compared with other studies (Table S1, Supporting Information). Therefore, the high extinction coefficient, excellent photothermal stability, high NIR-II photothermal conversion efficiency, and excellent biocompatibility of Pdots enabled their outstanding performance for dual-model PA/FL imaging-guided PTT.

### 2.4 In Vitro Biocompatibility CPdots

**Figure 4:**
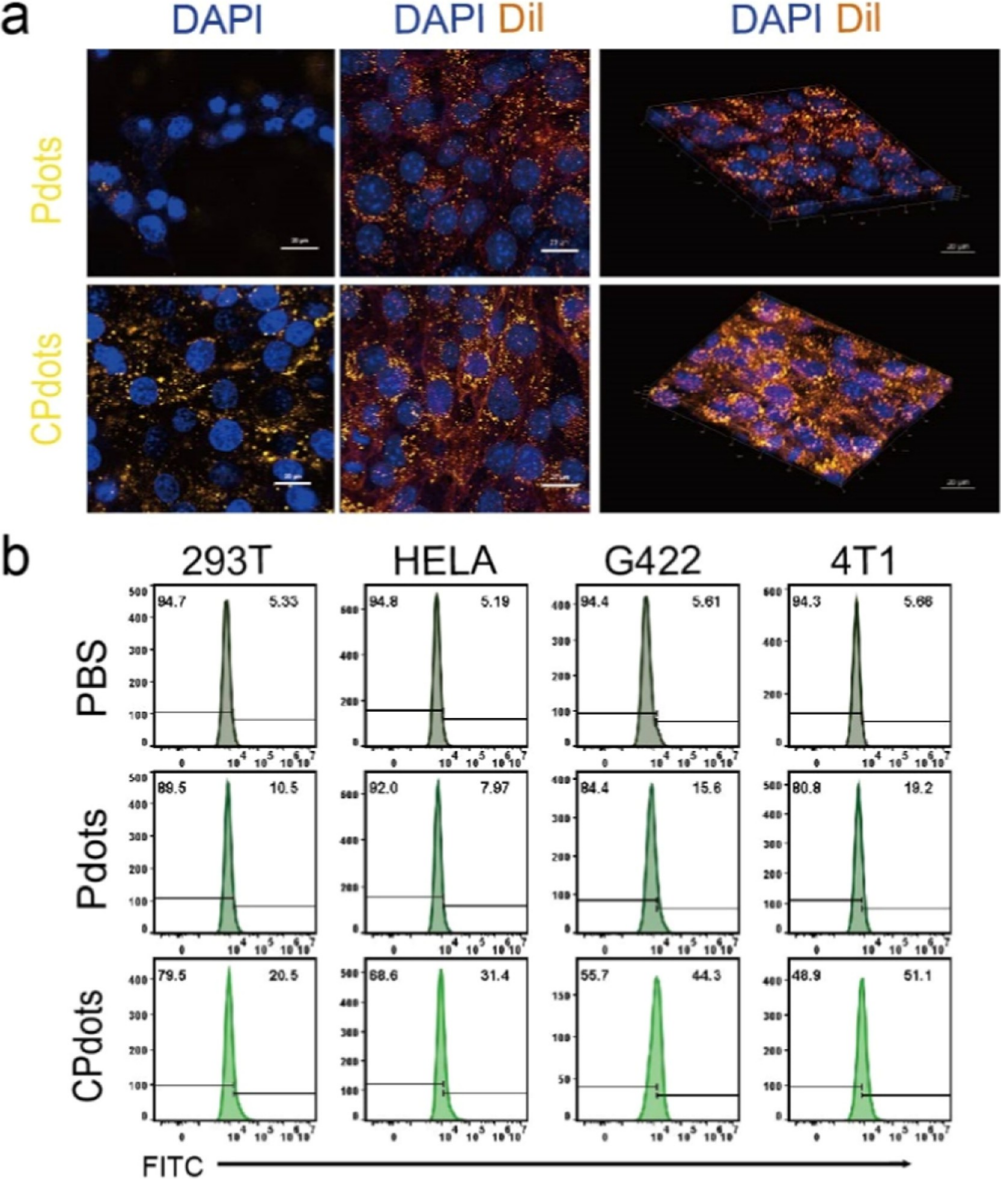
(a) Confocal imaging of 4T1 cancer cells after incubation with *m*-PBTQ4F Pdots and CPdots (Pdots, CPdots = 25 μg/mL) for 12 h, respectively. Yellow fluorescence denoted the nanoparticles, whereas cell nuclei (blue) were stained with 4′,6-diamidino-2-phenylindole (DAPI) (scale bar: 20 μm), and CM (orange) was stained with dil. (b) Flow cytometry analysis of *m*-PBTQ4F Pdots and CPdots incubation with 293T, HELA, G422, and 4T1 (Pdots, CPdots = 25 μg/mL).

To access the biocompatibility of *m*-PBTQ4F Pdots and CPdots, the cell uptake of biomimetic *m*-PBTQ4F Pdots was inspected by confocal imaging (Figure 4a). Due to the lack of a NIR-II confocal imaging system, (*m*-PBTQ4F)-(CN-PPV) Pdots (CNPdots) were produced through the doping of NIR-II emitting polymer (*m*-PBTQ4F) with orange-emitting polymer (CN-PPV). CNPdots were able to offer fluence emission under 488 nm laser irradiation. The absorption and excitation spectra of CNPdots are provided in Figures S4 and S5. To further verify the targeting ability of CPdots, we cocultured Pdots and CPdots with four different cell lines and performed flow cytometry analysis. As shown in Figure 4b, the homologous 4T1 cells exhibited the highest uptake of CPdots. One limitation of this work is that we are unsure whether the cells are membrane-bound or internalized.

**Figure 5:**
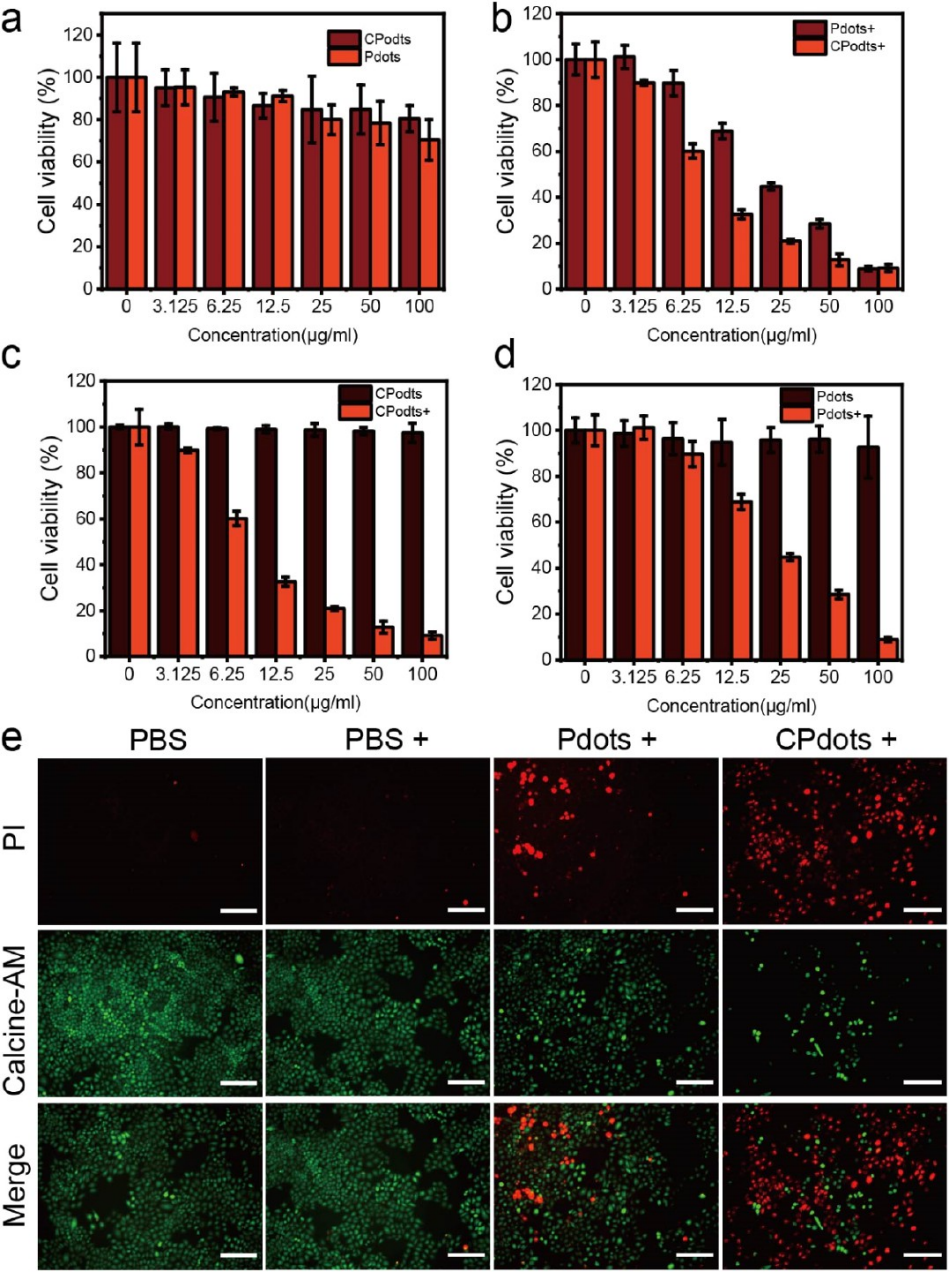
(a) CCK8 assay of 293T cells treated with *m*-PBTQ4F Pdots and CPdots at various concentrations for 24 h to assess cell viability. Error bars denote the standard deviation (*n* = 3). (b) Cell viability assessment of 4T1 cells after incubation with various concentrations of *m*-PBTQ4F Pdots and CPdots (+ = 0.5 W/cm^2^ 6 min of 980 nm laser). Error bars indicate standard deviation (*n* = 3). (c) Cell viability assessment of 4T1 cells after incubation with various concentrations of *m*-PBTQ4F Pdots (+ = 0.5 W/cm^2^ 6 min of 980 nm laser). Error bars indicate standard deviation (*n* = 3). (d) Cell viability assessment of 4T1 cells after incubation with various concentrations of CPdots (+ = 0.5 W/cm^2^ 6 min of 980 nm laser). Error bars indicate standard deviation (*n* = 3). (e) Fluorescence microscopy imaging of live/dead HeLa cells incubated with *m*-PBTQ4F Pdots and CPdots (Pdots, CPdots = 25 μg/mL) for 12 h, followed by 980 nm laser irradiation at 0.5 W/cm^2^ for 10 min (scale bar: 200 μm).

In addition, a 5-(2,4-disulfophenyl)-3-(2-methoxy-4-nitrophenyl)-2-(4-nitrophenyl)-2*H*-tetrazolium (WST-8) cell counting kit (CCK-8) was utilized to inspect the biocompatibility and efficiency of killing cancer cells of Pdots. The results demonstrated that *m*-PBTQ4F Pdots and CPdots showed no obvious toxicity effects on 4T1 cells after incubation for 24 h, even at a high concentration of 100 μg/mL (Figure 5a). To further assess the efficacy of PTT on living cells, the photothermal ablation of 4T1 and HeLa cancer cells was performed by using both the CCK-8 assay and fluorescence imaging. Under 980 nm laser irradiation, various concentrations of nanoparticles as photothermal agents were incubated with the cancer cells. The CCK-8 results illustrated that the cell viabilities significantly reduced with an increased concentration of nanoparticles. For example, approximately 80% of the cancer cells were killed with a low concentration (6.25 μg/mL) of CPdots, whereas approximately 80% of the cancer cells were dead with 25 μg/mL *m*-PBTQ4F Pdots with the laser irritation at a power density of 0.5 W/cm^2^ (Figure 5b). Therefore, the viability of cancer cells under PTT showed a photothermal agent concentration-dependent property. In addition, fluorescence microscopy imaging of calcein acetoxymethyl ester (calcein-AM)/propidium iodide costained HeLa cells confirmed the high PTT ablation efficacy of CPdots (Figure 5c), demonstrating that CPdots can serve as an excellent photothermal agent for enhanced PTT in the presence of 980 nm laser irradiation at a very low power density.

### 2.5 Dual-Modal PA and Fluorescence Imaging Performance of CPdots in the NIR-II Window

**Figure 6:**
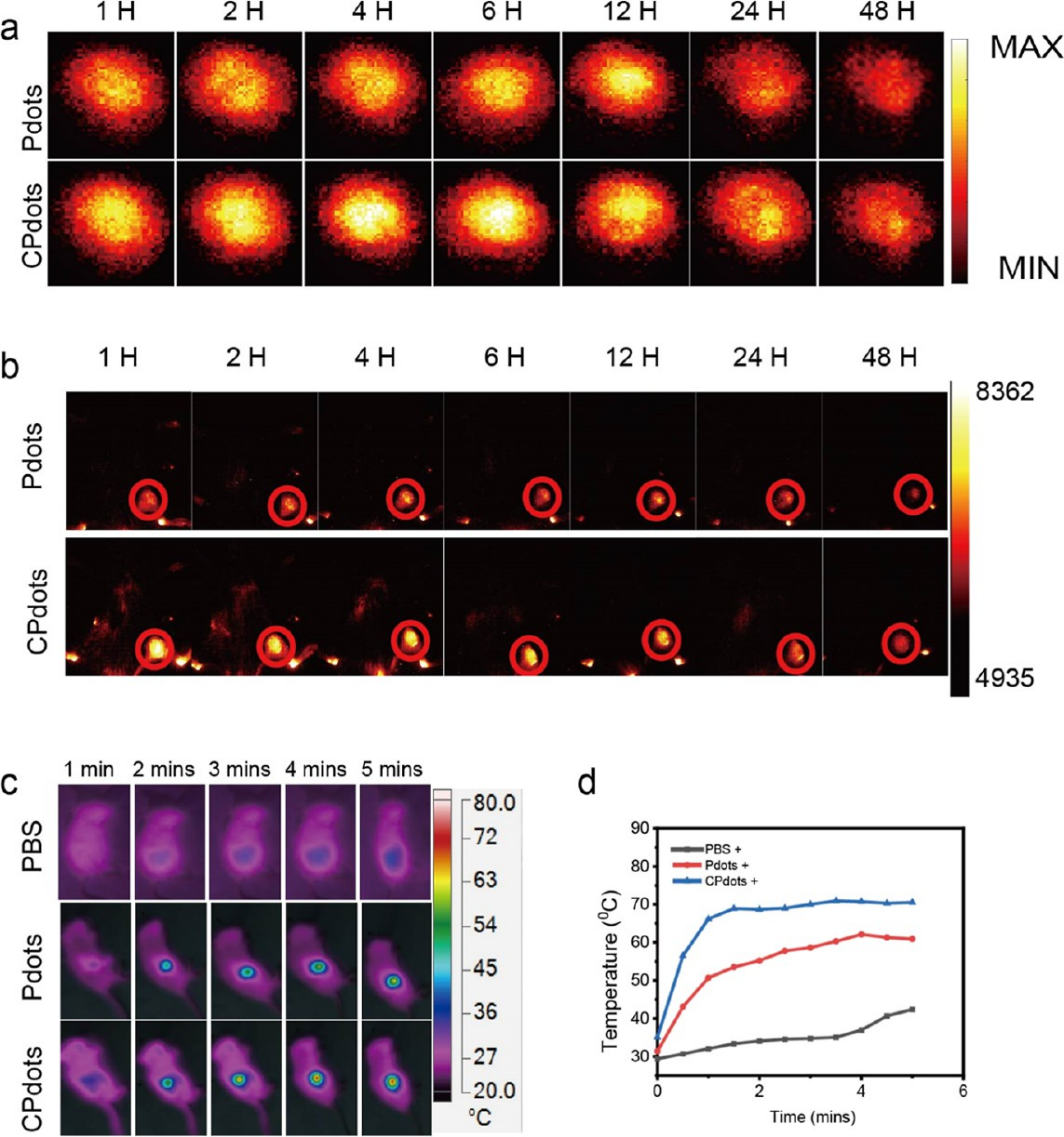
(a) In vivo PAI of 4T1 tumor-bearing mice after intravenous (i.v.) injection of *m*-PBTQ4F Pdots and CPdots (at a dose of 2.0 mg/kg) at different time points (1, 2, 4, 6, 12, 24, and 48 h). (b) In vivo FL imaging of 4T1 tumor-bearing mice after intravenous (i.v.) injection of *m*-PBTQ4F Pdots and CPdots (at a dose of 2.0 mg/kg) at different time points (1, 2, 4, 6, 12, 24, and 48 h). (c) IR thermal images of 4T1 tumor-bearing mice under NIR 980 nm laser irradiation with a laser power of 0.5 W/cm^2^ for 5 min. Laser irradiation was performed 12 h after intravenous (i.v.) injection of phosphate-buffered saline (PBS), *m*-PBTQ4F Pdots, and CPdots (at a dose of 2.0 mg/kg). (d) Mean tumor temperature as a function of laser irradiation time after intravenous (i.v.) injection of PBS, *m*-PBTQ4F Pdots, and CPdots. Here, CPdots denote the *m*-PBTQ4F CPdots.

In addition, in vivo, PA and FL imaging was carried out in the NIR-II window to visualize the increased accumulation and homologous targeting capability of CPdots as compared to those of *m*-PBTQ4F Pdots. In particular, a 4T1 tumor-bearing BALB/c mouse model was established and then the mice were intravenously administrated with *m*-PBTQ4F Pdots, and the PA signal intensity at the tumor site was monitored at different time points by using our homemade NIR-II PAI system (Figure S6, Supporting Information). Figure 6a shows a plot of generated in vivo PA images and corresponding PA signal intensities at the tumor site after the iv injection of nanoparticles under 980 nm laser irradiation at different time points. It was discovered that *m*-PBTQ4F Pdots could accumulate at the tumor site through the EPR effect and homologous targeting. In particular, PA signals were detected at 1 h postinjection of CPdots, which then gradually increased until they reached the peak at 6 h postinjection. The PA signal intensities began to decrease until they became stable at 48 h postinjection. For comparison, in vivo PA imaging was also performed with *m*-PBTQ4F Pdots as the contrast agent. It was discovered that the PA signal intensity reached the peak at 12 h postinjection of *m*-PBTQ4F Pdots and then began to decrease slowly until it became feeble at 24 h postinjection. One limitation of the PA imaging is that we did not image the animals at baseline (preinjection), making estimations of the signal amplification impossible.

In addition, for in vivo NIR-II FL imaging, obvious fluorescence signals were observed at the tumor site at 1 h postinjection of CPdots, and then the fluorescence intensities gradually increased over time until they reached the peak at 6 h postinjection (Figure 6b). More importantly, CPdots exhibited stronger fluorescence signals at the tumor sites as compared with the Pdots at each time point (Figure S7, Supporting Information). The higher NIR-II FL and PA signals in the tumor regions for CPdot-injected mice can be attributed to the improved tumor accumulation of CPdots since *m*-PBTQ4F Pdots and CPdots demonstrated the same absorption and fluorescence intensities at the same concentration. Herein, CPdots exhibited the advantages of shorter tumor enrichment time and enhanced NIR-II PAI and FL imaging capabilities as compared with *m*-PBTQ4F Pdots.

Further, the tumor sites of mice were exposed to 980 nm laser irritation with 0.5 W/cm^2^ for 5 min at 12 h postinjection of various Pdots. The temperature evolution under PTT was imaged using an infrared thermal imager, while that of the control groups injected with PBS was also monitored and measured (Figure 6c). As shown in the photothermal images, the temperature of tumor sites injected with CPdots quickly reached 65 °C in 5 min, which is sufficient to ablate tumors. However, the temperature of tumor sites injected with PBS only increased to 38 °C in 5 min under the same irradiation conditions (Figure 6d), demonstrating the low treatment efficacy.

### 2.6 In Vivo NIR-II PTT of Tumors with CPdots

**Figure 7:**
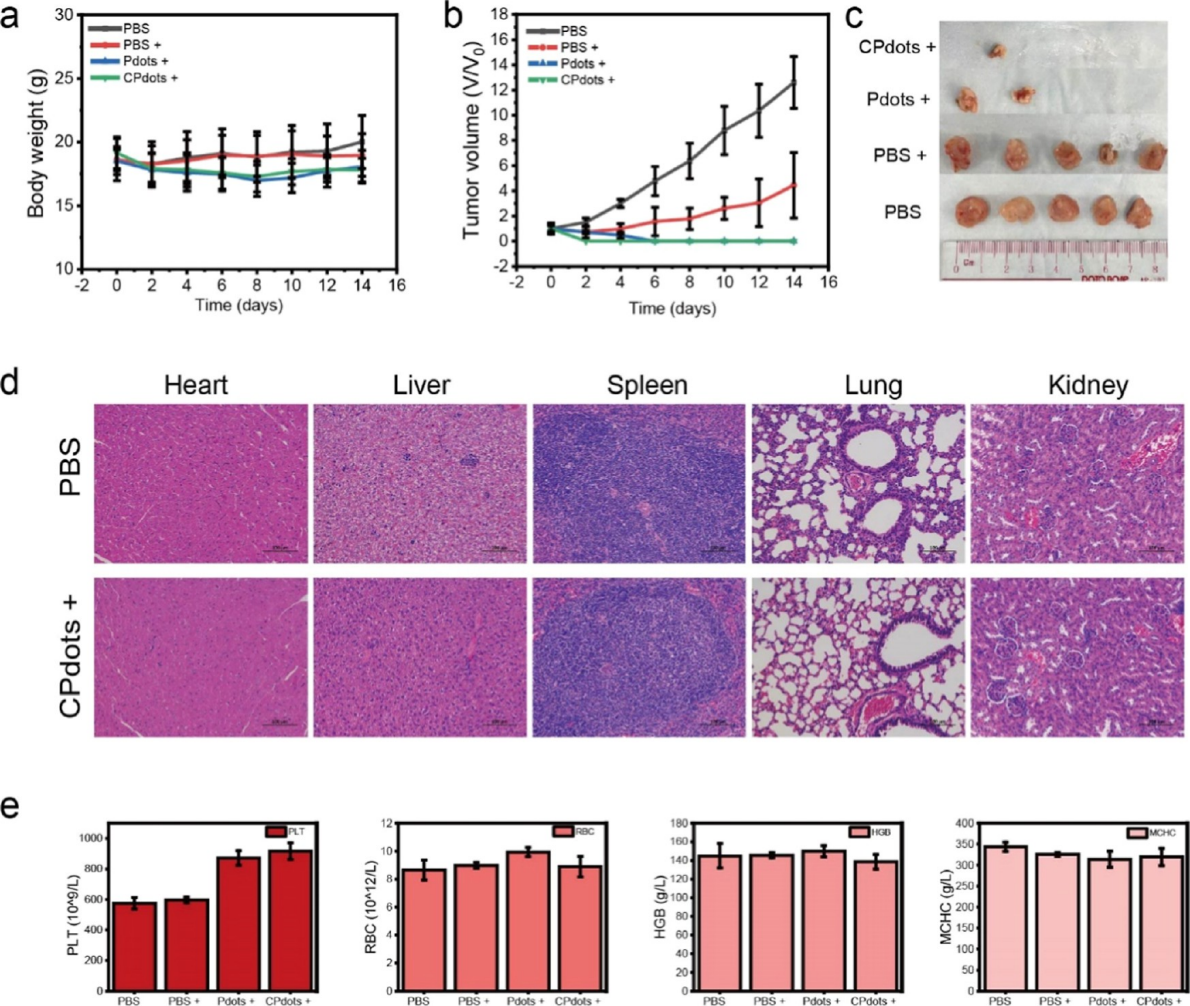
(a) Body weight changes of mice after various treatments over 14 days. Each data point represents the mean ± SD from *n* = 5 animals. (b) Relative tumor volume curves. Each data point denotes the mean ± SD from *n* = 5 animals. (c) Photographs of tumors after excision from the mice after different treatments. (i) PBS; (ii) PBS+; (iii) *m*-PBTQ4F Pdots+; and (iv) CPdots+, (+ = 980 nm, 0.5 W/cm^2^, 5 min). (d) Hematoxylin and eosin (H&E) staining images of major organs from normal control mice and treated mice (CPdots+) after treatment. (e) Blood routine indexes change over different treatments. RBC: red blood cell, PLT: platelet, MCH: mean corpuscular hemoglobin, MCHC: mean corpuscular hemoglobin concentration. Each data value denotes the mean ± SD from *n* = 3 animals.

The efficacy of PTT was also inspected using the same mice model. All procedures were performed in compliance with the Animal Care and Use Committee of the University of Macau. BALB/c mice with tumor sizes of approximately 100 mm^3^ were randomly divided into four groups (*n* = 5 per group), before i.v. injection of CPdots and Pdots at a dose of 2.0 mg kg^–1^ or the same volume of PBS. As shown in Figure 7a, the body weight changes showed no obvious difference during the 14 day treatment for all treatment groups, indicating the low toxicity of CPdots and *m*-PBTQ4F Pdots under NIR-II laser irradiation. The tumor sizes of the four groups were successfully measured every 2 days (Figure 7b). It was discovered that both PBTQ4F Pdots and CPdots inhibit tumor growth under laser irradiation. However, the majority of tumor volumes were eliminated for the CPdots-based PTT treatment group, showing no recurrence within 2 weeks (Figure 7c). However, this is not the case for the control groups, showing no tumor growth inhibition. Therefore, our findings demonstrated that CPdots exhibited an excellent PTT effect for killing cancer cells in vivo.

The long-term biodistribution and toxicity of nanoagents remain one of the most important concerns for their potential clinical applications. In this study, a histological examination was performed by hematoxylin and eosin (H&E) of the major organ slices (including the heart, liver, spleen, lung, and kidney) of tumor-bearing mice after the treatment. As shown in Figure 7d, the treated mice exhibited no appreciable damage to normal tissues, indicating that CPdots and *m*-PBTQ4F Pdots showed no observable side effect or toxicity to normal tissues with the tested dose as compared with the negative control (Figure S8, Supporting Information). In addition, body clearance experiments were performed to inspect the in vivo kinetics and biodistribution of both the CPdots and *m*-PBTQ4F Pdots. As a result, CPdots and *m*-PBTQ4F Pdots were intravenously injected into the BALB/c mice via the tail vein, respectively. Then, mouse blood samples were collected and the major organs including the brain, heart, lung, liver, spleen, and kidneys were excised and well visualized at 48 h postinjection by fluorescence microscopy imaging (Figure S9, Supporting Information). It was discovered that the strong fluorescence signals of CPdots and *m*-PBTQ4F Pdots were mainly detected in the liver and spleen, whereas the CPdots group showed much brighter FL signals. Further, we also inspected the toxicity of CPdots in blood, in which the tumor-bearing mice were intravenously injected with CPdots and *m*-PBTQ4F Pdots and blood samples were collected 14 days postinjection. Meanwhile, the routine blood indices of the CPdot-treated mice were measured and compared to those of the untreated groups, as displayed in Figure 7e. The blood routine indices included red blood cells, hemoglobin (HGB), platelets (PLT), and mean corpuscular hemoglobin concentration (Figure S10, Supporting Information). All detection indices were normal for the treated group, demonstrating that iv injection of CPdots or *m*-PBTQ4F Pdots has no major hepatotoxic effects or renal toxicity.

## 3 Conclusions

In this study, we developed biomimetic CPdots that were able to specifically target the tumor for enhanced dual-modal imaging-guided cancer PTT in the NIR-II window. CPdots were prepared through coextrusion of *m*-PBTQ4F Pdots and 4T1 cell membranes, whereas cell membrane camouflage did not compromise their NIR-II fluorescence, NIR-II PA, and NIR-II photothermal properties. Through the homologous targeting mechanism, CPdots were able to preferentially target 4T1 tumors, resulting in higher accumulation in tumor tissues than *m*-PBTQ4F Pdots. As such, CPdots can serve as the optimal PTT nanoagents to provide amplified NIR-II fluorescence and PA signals for tumor detection and enhance tumor phototherapeutic efficiency. In addition, both blood circulation studies and histological examinations demonstrated that CPdots showed no toxicity to the mice during the entire treatment, thereby avoiding unnecessary side effects. Therefore, CPdots exhibited great promise for precise diagnosis and localization of tumor sites to guide efficient PTT. To further validate the biocompatibility of the material and the effects of the laser, it is essential to include a no-light control group in future studies. Finally, further studies on pharmacokinetics will provide additional insights into the entire field of materials and warrant further exploration.

In summary, a new strategy was proposed to construct cell membrane-coated biomimetic Pdot for targeted cancer theranostics. The developed theranostic nanoplatforms that incorporate enhanced diagnostic and therapeutic capability can confer upon these superior nanoprobes vast potential for clinical translation.

## 4 Materials and Experimental Procedures

### 4.1 Materials

The semiconducting polymer poly [2-methoxy-5(2-ethylhexyloxy)-1,4-(1-cyanovinylene1,4-phenylene)] (CN-PPV, average molecular weight: 25,000, polydispersity: 2.1) was purchased from American Dye Source, Inc. (Quebec, Canada). The semiconducting polymer poly[4-(4,8-bis(5-(2-ethylhexyl)thiophen-2-yl)benzo[1,2-*b*:4,5-*b*′]dithiophen-2-yl)-2-(2-ethylhexyl)-6,7-bis(3-((2-ethylhexyl)oxy)-4,5-difluorophenyl)-2*H*-[1,2,3]triazolo[4,5-*g*]quinoxaline](PBTQ4F, average molecular weight: 30.2 kDa, polydispersity: 1.38) was synthesized by Prof. Wu’s group^20^ (BME SUSTech, China). The functional polymer, polystyrene graft ethylene oxide functionalized with carboxyl groups (PS–PEG), was purchased from Polymer Source Inc. (Quebec, Canada). PBS, Dulbecco’s modified eagle medium (DMEM), penicillin/streptomycin, fetal bovine serum (FBS), and trypsin–EDTA were obtained from Thermo Fisher Scientific. All materials were used without further purification, unless otherwise indicated. Tetrahydrofuran (THF) was used to prepare polymer dots (Pdots). Ultrapure H_2_O (18.25 MΩ·cm^–2^ at 25 °C) was used throughout the study, and all other chemical reagents were used as received.

### 4.2 Characterization

TEM images were captured by a JEM1200EX TEM (JEOL, Japan). Diameter and ζ potential were measured on a Malvern Nano-ZS particle sizer (Malvern Instruments, Southborough, UK). Fluorescence spectra were obtained from a HORIBA Scientific Fluorolog-3 Fluorescence Spectrometer (HORIBA Scientific, United States). Absorption spectra were obtained from a Shimadzu UV-1800 ultraviolet–visible spectrophotometer (Cole-Parmer, United States). In vitro and in vivo PA signal measurement was performed on our homemade multispectral PA imaging system. In vivo confocal fluorescence imaging was carried out on a Carl Zeiss LSM710 Confocal (Carl Zeiss AG, German). In vitro fluorescence imaging was carried out on Raptor Photonics Ninox 640 SU NIR CCD from Prof. Qu’s group (IAPME UM, China).

### 4.3 Cell Culture

HeLa cervical cancer cells and 293T and 4T1 mammary carcinoma cells were cultured at 37 °C in DMED containing 10% FBS and 1% penicillin/streptomycin with a humidified environment containing 5% CO_2_. For in vitro experiments, cells are cultured in T25 flasks for 24 h and then digested and seeded at 5000–20,000 cells per well in 96-well or 48-well plates. After 24 h, nanomaterials are added, and the samples are incubated for an additional 12 h before treatment. For in vivo treatments, T75 flasks are used, and cells are digested for experimentation when they reach 80–90% confluency. To extract cell membranes, T175 flasks are used, and cells are digested for experimentation when they reached 80–90% confluency.

### 4.4 Preparation of Pdots and CNPdots

The Pdots were prepared by using a reprecipitation method as described previously. PBTQ4F (0.1 mg) and PS–PEG–COOH (0.1 mg) were dissolved into a THF (1 mL) solution. The mixture was then quickly injected into 10 mL solutions (9 mL of Milli-Q water + 1 mL of 0.025% Trixon-X100) under vigorous sonication. The THF was removed, and the solution was concentrated by rotary evaporation. A small fraction of aggregated Pdots were removed by filtration through a 0.22 μm membrane filter. The CNPdots were also prepared by using a reprecipitation method. CN-PPV(0.1 mg), PBTQ4F(0.1 mg), and PS–PEG–COOH (0.2 mg) were dissolved into a THF (2 mL) solution. The mixture was then quickly injected into 10 mL solutions (9 mL Milli-Q water + 1 mL 0.025% Trixon-X100) under vigorous sonication. The THF was removed, and the solution was concentrated by rotary evaporation. A small fraction of aggregated Pdots were removed by filtration through a 0.22 μm membrane filter. The concentrations of solutions were then determined using a UV–vis spectrophotometer.

### 4.5 Preparation of the Cancer Cell Membrane

To obtain the 4T1 cancer cell membrane, we used a Membrane and Cytosol Protein Extraction Kit (Beyotime, China). The membrane protein extraction reagents A and B were dissolved and mixed at room temperature and then immediately placed on ice. The necessary amounts of reagents A and B were prepared, and PMSF was added just minutes before use, aiming for a final concentration of 1 mM. Approximately 20 to 50 million cells were cultivated and washed once with PBS, and the cells were scraped off with a scraper and collected using a pipet. They were centrifuged to collect the cells, the supernatant was removed, and the cell pellet was kept aside for further use. 1 mL of membrane protein extraction reagent A was added, which has been supplemented with PMSF, to the 20 to 50 million cells, and the cells were gently and thoroughly resuspended and incubated on ice for 10 to 15 min. They were frozen and thawed twice, consecutively in liquid nitrogen and at room temperature. Then, a small sample was taken, and the samples were inspected under a microscope to confirm that more than 70% of the cells were lysed. To remove nuclei and unbroken cells, they were centrifuged at 700*g* at 4 °C for 10 min, and the supernatant was carefully collected into a new centrifuge tube. The supernatant was centrifuged at 14,000*g* at 4 °C for 30 min to pellet the membrane fragments. 200 μL of membrane protein extraction reagent B was added, vortexed at high speed for 5 s to resuspend the pellet, and incubated on ice for 5 to 10 min. The vortexing and ice incubation steps were repeated 1 to 2 times to completely extract the membrane proteins. Finally, centrifuging was done at 14,000*g* at 4 °C for 5 min, and the supernatant was collected as the final membrane protein solution. It was stored at −70 °C for future use.

### 4.6 Preparation of CPdots

To coat cell membranes onto the surface of Pdots, cancer cell membranes (1.0 mg/mL) were mixed with Pdots solution (1.0 mg/mL), and the mixture was sonicated for 10 min. The excess membrane fragments were removed by centrifugation, and the obtained CPdots were stored at 4 °C for further use.

### 4.7 Agarose Gel Electrophoresis

Agarose powder was dissolved in 1× tris/boric acid/ethylenediaminetetraacetic acid (TBE) buffer and used to prepare agarose gels (2%). Cancer cell membrane, Pdots, CPdots, and stander maker (each 300 μL) were mixed with the 10 μL 4× Laemmli sample buffer, then heated at 95 °C for 5 min, and cooled in an ice bath. Afterward, 20.0 μL of the prepared sample was loaded into each well of 10.0% SDS–PAGE gels and run at 180 V for 20 min. The obtained gels were subsequently stained with Coomassie Blue for 10 min 90 rpm and then washed overnight to visualize protein bands.

### 4.8 In Vitro Cytotoxicity Assay

4T1 mammary carcinoma cells and 293T cells cultured in 96-well plates were incubated with Pdots and CPdots at different concentrations for 24 h. Then the cells were cultured in DMEM containing 10.0% CCK-8 for another 1 h. To calculate the cell viability, the absorbance of the cell culture medium was measured using a microplate reader.

### 4.9 In Vitro Cellular Uptake Assay

4T1 mammary carcinoma cells, 293T cells, GL261 cells, and Hela cells cultured in confocal cell culture dishes (4 × 10^4^ cells/dish) were incubated in DMEM containing Pdots and CPdots at the concentration of 25 μg/mL for 12 h. The treated cells were washed, fixed, and then stained with DAPI.

### 4.10 Animals and Tumor Models

All procedures were performed in compliance with the Animal Care and Use Committee of the University of Macau. DMEM (100 μL) containing 1 × 10^6^ 4T1 cells was subcutaneously implanted into the right side of the back of 6 week old female Balb/C mice to establish the xenografted tumor models. 4T1 tumor bearing mice were used for fluorescence and PA imaging and cancer therapy experiments after 7 days of growth.

### 4.11 In Vivo Cancer Therapy

4T1 tumor bearing mice were intravenously injected with PBS, Pdots, and CPdots (at a dose of 2.0 mg/kg, *n* = 5 animals). At the 12 h postinjection time point, the tumors of mice were irradiated using an 980 nm laser for 5 min (0.5 W/cm^2^), and an IR thermal camera was used to record the tumor temperature changes every 30 s. To monitor the therapeutic efficiency and biosafety, tumor sizes and body weights of mice were measured every other day for 14 days. The tumor volume was calculated as follows: *V* = (length) × (width)^2^/2. Relative tumor volume was calculated as *V*/*V*_0_ (*V*_0_ was the initial tumor volume).

### 4.12 Histopathological Evaluation

For histology analysis, the harvested organs were fixed in 4% neutral buffered paraformaldehyde and embedded with paraffin. Sections of the main organs of interest of the mice were stained with H&E. The histological sections were imaged using an optical.

## Data Availability

The data sets used and/or analyzed during the current study are available from the corresponding author on reasonable request.

## Supporting Information Available

The preparation procedures of Pdots and CPdots, the stability results and fluorescence intensity data of the materials, cooling curve of the material, characterization of CNPdots material, schematic of the homemade multispectral PA imaging system, fluorescence intensity data of animal tumor regions and H&E-stained sections, fluorescence images of animal viscera zones, hematology results of animals, comparison table of material PCE, and the PCE calculation process (PDF)
