# Supplementary material for: 4T1 Cell Membrane-Coated Pdots with NIR-II Absorption and Fluorescence Properties for Targeted Phototheranostics of Breast Tumors
Source: ACS Appl Mater Interfaces. 2024 Nov 21;16(48):66425–35. doi: 10.1021/acsami.4c12845 (PMC11622192; doi:10.1021/acsami.4c12845)
Supplement: Supplementary file 1 — am4c12845_si_001.pdf [file am4c12845_si_001.pdf]

Supporting Information

4T1 Cell Membrane-Coated Pdots with NIR-II Absorption and Fluoresces Properties for Targeted Phototheranostics of Breast Tumors

Jintong Guo[1,2,#], Ye Liu[3,#], Xiao Liang[1], Zhiyi Chen[4,*], Bin Liu[5,*]， Zhen Yuan[1,2,*]

[1]Faculty of Health Sciences, University of Macau, Macau SAR 99999, China

[2]Centre for Cognitive and Brain Sciences, University of Macau, Macau SAR 99999, China

[3]Department of Biomedical Engineering, Southern University of Science and Technology, Shenzhen, Guangdong 518055, China

[4] The Affiliated Changsha Central Hospital, Hengyang Medical School, University of South China, Changsha 410004, Hunan, China

[5] Zhujiang Hospital of Southern Medical University, Guangzhou 510280, Guangdong, China

*Corresponding Author, Prof. Zhen Yuan, Email: zhenyuan@um.edu.mo; Prof. Bin Liu, nysylb@163.com; Prof. Zhiyi Chen, Email: zhiyi_chen@usc.edu.cn

[#]These authors contributed equally to this work.

1. Figures

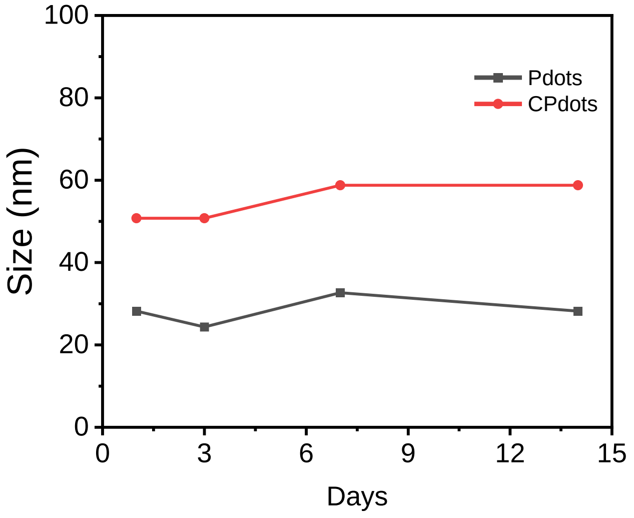

Figure S1. Determination of nanomaterial size at different time points.

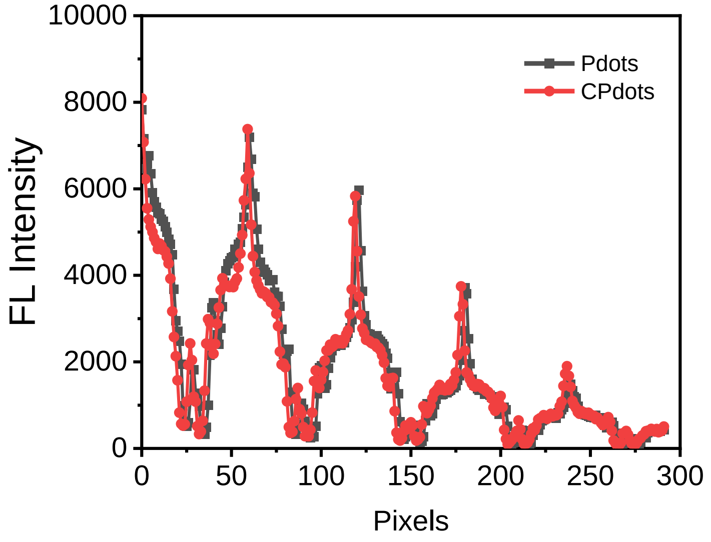

Figure S2. Fluorescence signal intensity of nanomaterials, with each peak representing the strongest signal in that region.

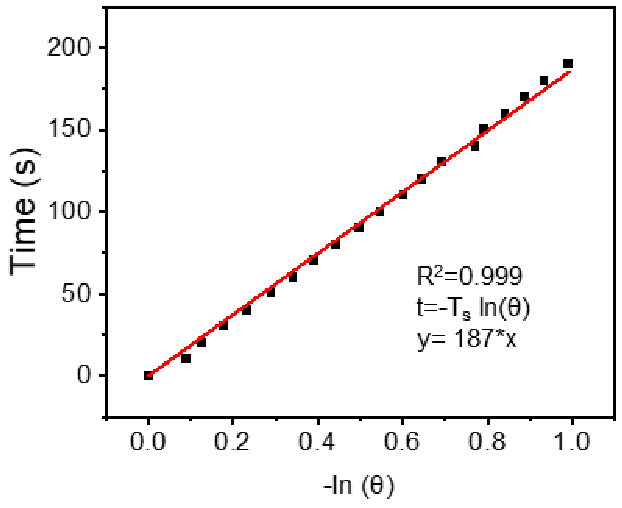

Figure S3. Linear time data versus −ln θ obtained from the cooling period.

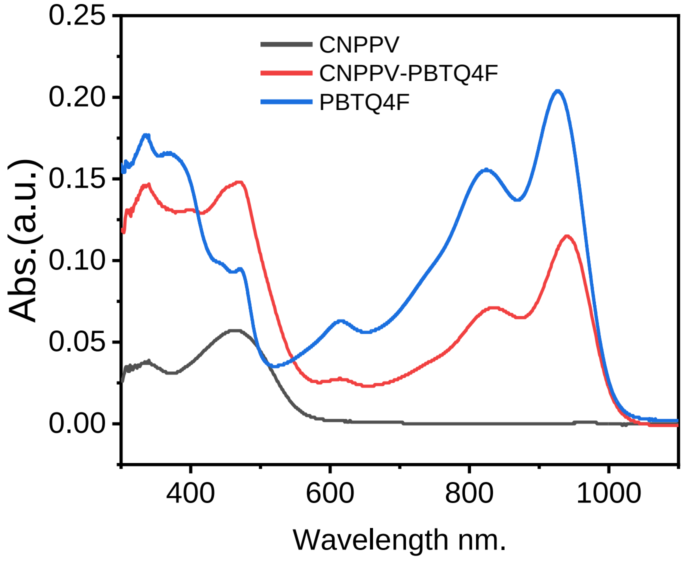

Figure S4. UV−vis absorption spectra of CNPdots.

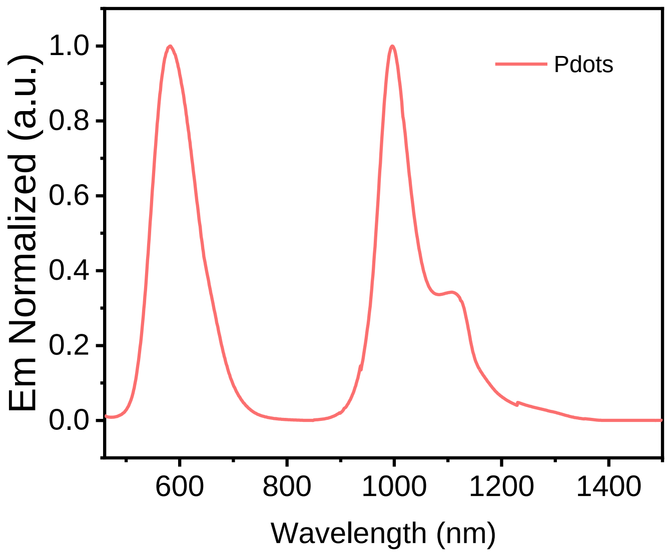

Figure S5. The fluorescence spectra of CNPdots.

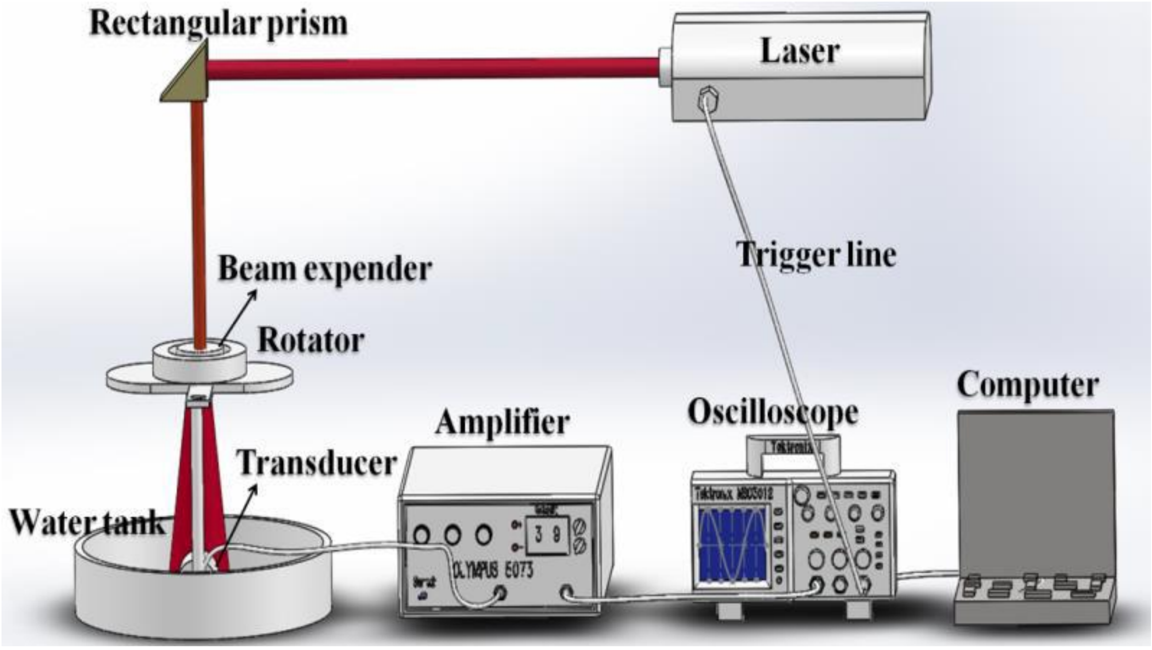

Figure S6. Schematic of our home-made multispectral photoacoustic imaging system.

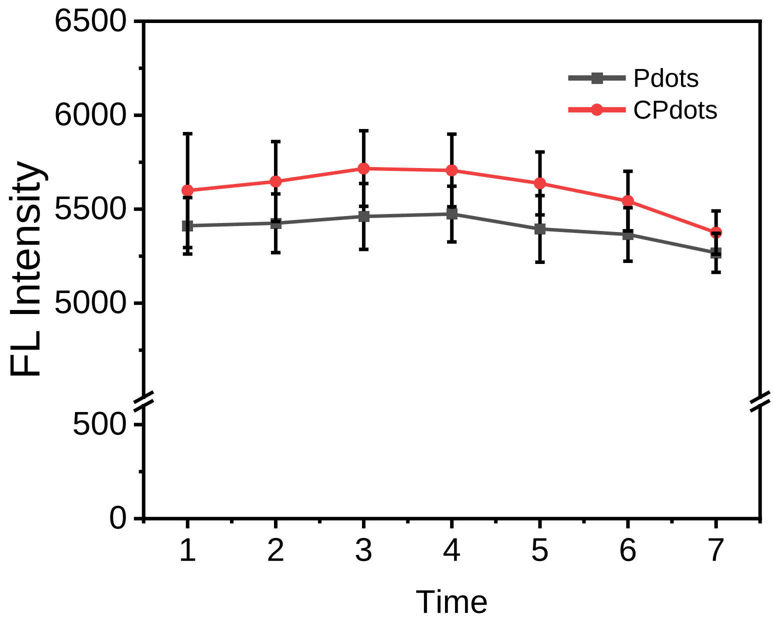

Figure S7. Fluorescence signal intensity in the tumor region at different time points after drug.injection.

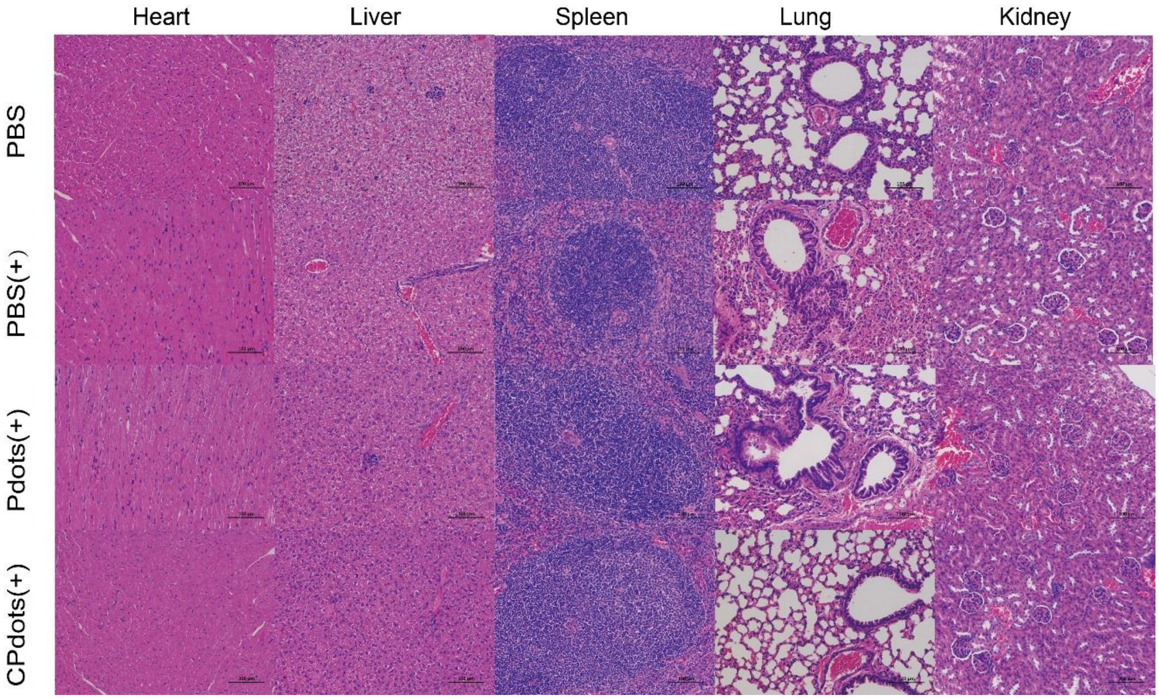

Figure S8. H&E stanning of the primary organs after photothermal therapy. H&E stanning studies of the liver, kidney, heart, spleen, and lung for PBS, PBS+ Laser, Pdots+ Laser and CPdots+

Laser treatment groups after 14 days treatment with the irradiation from the NIR light (980-nm, 0.5W/ cm^2).

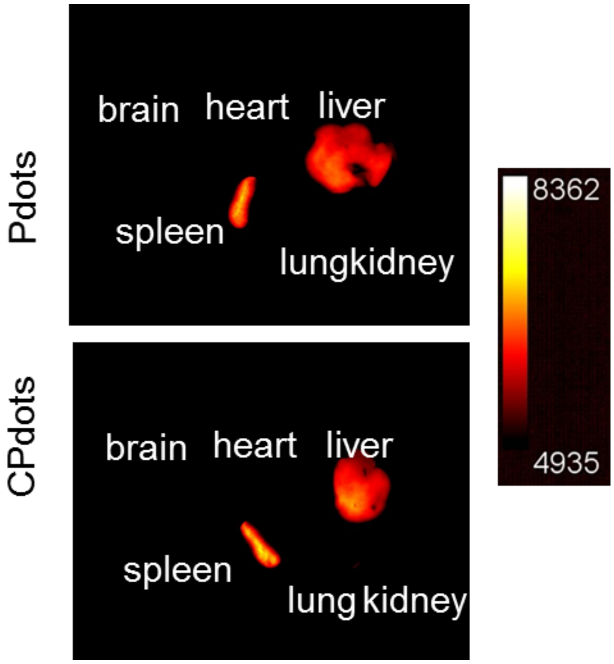

Figure S9. NIR- fluorescence imaging of various organs (from the right to left and top to bottom: the brain, heart, liver, spleen, lung and kidneys) dissected from mice after the i.v. injection of CPdots and Pdots (at a dose of 2.0 mg/kg) at 48 h post-inject.

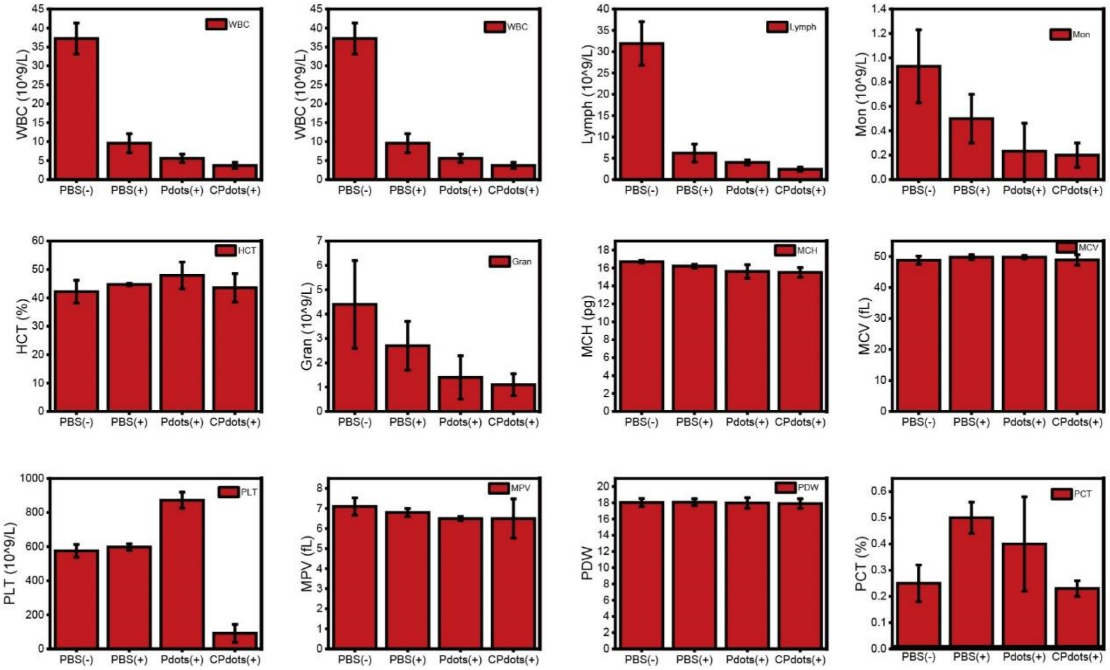

Figure S10. The blood routine indexes change over different treatments. Each data value denotes the mean ± SD from *n* = 3 animals.

2.Table

NIR-II NPs	FE-T NPs[1]	TTQ-2TC NPs[2]	PTTe NPs[3]	ADPPTN[4]	FPS-PVP NSs[5]	AuNRs@SiO_2–RB@MnO_2[6]
PCE	43.9%	34.9%	47.5%	44%	43.3%	54%

Table S1. The photothermal conversion efficiency (PCE) of NIR-II nanoparticles(NPs).

3. Calculation: Photothermal conversion efficiency[7, 8]

To measure photothermal conversion efficiency, Pdots (OD=1) is added to a quartz cuvette. Then Pdots are exposed to 980 nm laser irradiation at a power density of 0.5 W/cm^2 to reach the equilibrium, followed by cooling down to room temperature. The photothermal conversion efficiency can be calculated using the following expressions. For an energy balance in a system, we can describe the total energy balance as:

$$\sum i\, m_i C_{p,i} \frac{dT}{dt} = \sum j\, Q_j = Q_l + Q_0 - Q_{ext} \quad \text{(S1)}$$

in which the i in $m_i C_{p,i}$ is the producs of mass and specific heat capacity of system components, T aggregate system temperature, and t time. The j energy term in Q_j contains the laser-induced energy source terms Q_l (from Pdots), Q_0 (from solvent and cuvette), and energy outputs Q_{ext}.

Q_l is the photothermal energy input from the Pdots, which is written,

$$Q_l = I(1 - 10^{-A_\lambda})\eta \quad \text{(S2)}$$

in which I is the incident laser power, A_λ is the optical density at laser wavelength (980 nm), and η is the photothermal conversion efficiency. Q_0 denotes the heat dissipated from optical absorbed by the solvent and container, which is measured independently using a container just containing aqueous samples without Pdots.

Q_{ext}, the external heat flux in the system, is nearly proportional to the linear thermal driving force with the heat-transfer coefficient *h* as the proportionality constant.

$$Q_{ext} = hA(T - T_{amb}) \quad \text{(S3)}$$

in which h and A denotes the heat transfer coefficient and surface area of the cuvette, respectively. hA can be determined by measuring the rate of temperature decrease after removing the light source. In the absence of any laser excitation, (Q_l + Q_0 = 0) substitute eq. (3) into eq. (1) , and we have,

$$\sum i\, m_i C_{p,i} \frac{dT}{dt} = -hA(T - T_{amb}) \quad \text{(S4)}$$

After the rearrangement and integration, the following expression for *t* is produced,

$$t = -(\frac{mC_p}{hA}) In(\frac{T_{amb} - T}{T_{amb} - T_{\max}}) \quad \text{(S5)}$$

in which m and C_p is the mass and heat capacity of water, respectively. To get the value of hA, a dimensionless driving force temperature, θ, is introduced, scaled using the maximum system temperature, $T_{\max}$, and a sample system time constant τ_s.

$$\theta = \frac{T_{amb} - T}{T_{amb} - T_{\max}} \quad \text{(S6)}$$

$$\tau_s = \frac{mC_p}{hA} \tag{S7}$$

in which τ_s is the slope of the linear time data from the cooling period,

$$t = -\tau_s \ln(\theta) \tag{S8}$$

Thus, the photothermal conversion efficiency (η) of Pdots is written,

$$\eta = \frac{hA(T_{\max} - T_{amb}) - Q_0}{I(1 - 10^{-A\lambda})} \tag{S9}$$

References

1. Yang, S., et al., *NIR-II Imaging-Guided Mitochondrial-Targeting Organic Nanoparticles for Multimodal Synergistic Tumor Therapy.* Small, 2023. **19**(26): p. 2207995.
2. Song, X., et al., *Conjugated Polymer Nanoparticles with Absorption beyond 1000 nm for NIR-II Fluorescence Imaging System Guided NIR-II Photothermal Therapy.* ACS Applied Polymer Materials, 2020. **2**(10): p. 4171-4179.
3. Wen, K., et al., *Achieving Efficient NIR-II Type-I Photosensitizers for Photodynamic/Photothermal Therapy upon Regulating Chalcogen Elements.* Advanced Materials, 2022. **34**(7): p. 2108146.
4. Wang, W., et al., *Semiconducting polymer nanoparticles for NIR-II fluorescence imaging-guided photothermal/thermodynamic combination therapy.* Biomaterials Science, 2022. **10**(3): p. 846-853.
5. Zhang, Q., et al., *Highly Efficient 2D NIR-II Photothermal Agent with Fenton Catalytic Activity for Cancer Synergistic Photothermal–Chemodynamic Therapy.* Advanced Science, 2020. **7**(7): p. 1902576.
6. Wen, C., et al., *NIR-II-responsive AuNRs@SiO₂–RB@MnO₂nanotheranostic for multimodal imaging-guided CDT/PTT synergistic cancer therapy.* Journal of Materials Chemistry B, 2022. **10**(22): p. 4274-4284.
7. Roper, D.K., W. Ahn, and M. Hoepfner, *Microscale Heat Transfer Transduced by Surface Plasmon Resonant Gold Nanoparticles.* The Journal of Physical Chemistry C, 2007. **111**(9): p. 3636-3641.
8. Chen, H., et al., *Highly absorbing multispectral near-infrared polymer nanoparticles from one conjugated backbone for photoacoustic imaging and photothermal therapy.* Biomaterials, 2017. **144**: p. 42-52.
